# Analytical Strategies for Fingerprinting of Antioxidants, Nutritional Substances, and Bioactive Compounds in Foodstuffs Based on High Performance Liquid Chromatography–Mass Spectrometry: An Overview

**DOI:** 10.3390/foods9121734

**Published:** 2020-11-25

**Authors:** Dario Donno, Maria Gabriella Mellano, Giovanni Gamba, Isidoro Riondato, Gabriele Loris Beccaro

**Affiliations:** Dipartimento di Scienze Agrarie, Forestali e Alimentari, Università degli Studi di Torino, 10095 Grugliasco, Italy; gabriella.mellano@unito.it (M.G.M.); giovanni.gamba@unito.it (G.G.); isidoro.riondato@unito.it (I.R.); gabriele.beccaro@unito.it (G.L.B.)

**Keywords:** food analysis, High Performance Liquid Chromatography–Mass Spectrometry (HPLC–MS) techniques, Orbitrap, High Resolution Mass Spectrometry (HRMS), natural substances, antioxidant molecules

## Abstract

New technology development and globalisation have led to extreme changes in the agri-food sector in recent years that need an important food supply chain characterisation from plant materials to commercial productions. Many analytical strategies are commonly utilised in the agri-food industry, often using complementary technologies with different purposes. Chromatography on-line coupled to mass spectrometry (MS) is one of the most selective and sensitive analytical methodologies. The purpose of this overview is to present the most recent MS-based techniques applied to food analysis. An entire section is dedicated to the recent applications of high-resolution MS. Covered topics include liquid (LC)– and gas chromatography (GC)–MS analysis of natural bioactive substances, including carbohydrates, flavonoids and related compounds, lipids, phenolic compounds, vitamins, and other different molecules in foodstuffs from the perspectives of food composition, food authenticity and food adulteration. The results represent an important contribution to the utilisation of GC–MS and LC–MS in the field of natural bioactive compound identification and quantification.

## 1. Introduction

New technology advancement and globalisation have led to extreme changes in the agri-food sector in recent years that need an important food supply chain characterisation from plant materials to commercial productions. Moreover, consumers call for more food assurances and information on geographical origin, safety, and quality of the used final products [1]. Testing should also be performed to gather additional information that regulatory agencies periodically require not only for a general evaluation of chemical composition but also to accurately identify and quantify all the compounds of interest. Given its importance, the characterisation of complex food matrices is becoming more and more essential [2].

**Figure 1:**
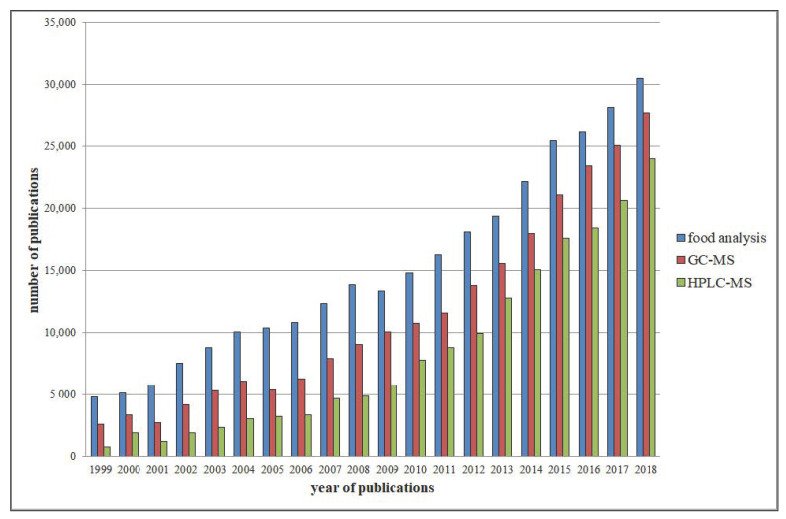
Evaluation of the manuscripts linked to food analysis, and those using GC–MS (20-fold) or HPLC–MS methods (50-fold). The *x*-axis represents the year of publications, while the *y*-axis represents the number of publications. Data derived from the Scopus database using the search term “food analysis” coupled (using “AND” as relationship term) with “HPLC–MS” and “GC–MS”, respectively. HPLC–MS = high performance liquid chromatography–mass spectrometry; GC–MS = gas chromatography–mass spectrometry.

Many analytical strategies are utilised in the agri-food industry, often using complementary technologies with different purposes. Chromatography on-line coupled to mass spectrometry (MS) or diode array detector (DAD) is one of the most selective and sensitive techniques in chemical analysis. Agri-food products are multicompound matrices; for this reason, sensitivity and selectivity are very important for the identification of components in trace [3]. Among the combinatorial approaches, high performance liquid chromatography–mass spectrometry (HPLC–MS) and gas chromatography–mass spectrometry (GC–MS) are the fastest-growing analytical strategies, already widely used in food analysis (Figure 1). In the last decade, the manuscripts describing GC–MS food analysis doubled, while papers that applied an HPLC–MS approach quadrupled.

**Figure 2:**
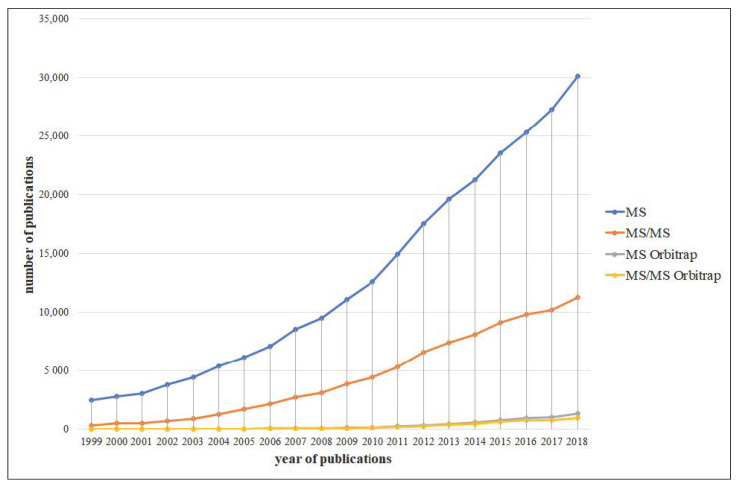
Evaluation of the manuscripts linked to food analysis together to (i) MS, (ii) MS/MS, (iii) MS Orbitrap, and (iv) MS/MS Orbitrap methods. The *x*-axis represents the year of publications, while the *y*-axis represents the number of publications. Data derived from the Scopus database using the search term “food analysis” coupled (using “AND” as relationship term) with “mass spectrometry”, “tandem mass spectrometry”, “mass spectrometry + Orbitrap”, and “tandem mass spectrometry + Orbitrap”, respectively. MS = mass spectrometry; MS/MS = tandem mass spectrometry.

In the last period (about 20 years), several important step-changes were developed in MS analysis. In LC–MS, the advances in ionisation interfaces (e.g., atmospheric pressure chemical ionisation—APCI and electrospray ionisation—ESI), coupled to the developments in MS instruments, allowed to apply LC–MS to the wider scientific area [4]. LC–MS techniques were surpassed by LC–tandem MS (MS/MS or MS^2^) ones, currently the standard approach, both for more sophisticated configurations and simple lab instruments. An important improvement has been also the improvement of high-resolution LC–MS technology (e.g., Orbitrap can reach resolutions of 10,000–200,000) and time-of-flight (ToF) instruments (as LC–ToF–MS in different configurations) [5,6,7] as shown in Figure 2.

The purpose of this overview is to present the most recent MS-based techniques applied in food analysis. An entire section is dedicated to the recent applications of high-resolution mass spectrometry (HRMS). Covered topics include liquid (LC)– and gas chromatography (GC)–MS analysis of natural bioactive substances. The applications of the GC/LC–MS techniques and relative interface systems in food analysis were described in this overview. The results represent an important contribution to the utilisation of GC–MS and LC–MS in the field of natural bioactive compound identification and quantification. The present review includes MS analysis of natural bioactive substances, including carbohydrates, flavonoids and related compounds, lipids, phenolic compounds, vitamins, and other different molecules in foodstuffs.

## 2. Analysis of Various Molecular Classes

Foods provide nutritional substances and phytochemicals useful for humans and their well-being and health conditions. Moreover, since the past, it was also known that specific foods ensure additional health-promoting benefits to people as prevention and/or treatment of several diseases [8]. For this reason, people achieved a better life quality by eating specific foods (e.g., meat, dairy products, fruits, vegetables, etc.) and relative derived-products (e.g., juices, jams, etc.) or taking dietary supplements or nutraceuticals. Moreover, regional, national, and international regulatory institutions have promoted intense research to identify and characterise new biologically active molecules to be used to formulate, develop, and improve new nutraceuticals and functional foods [9]. In the last years, companies, licensed professionals, marketers, and manufacturers have matured due to the increasing demands for bioactive substances, phytonutrients, nutraceuticals, and their therapeutic services [10]. These substances and compounds are secondary metabolites, namely molecules produced by the plants but not directly involved in the normal development, growth, or reproduction of a natural organism. They may act as “defence” molecules against biotic and abiotic stresses (e.g., parasites, diseases, ultraviolet radiation and oxidants, predators, etc.) for interspecies competition and to facilitate and improve the reproductive processes (e.g., they may also serve as colouring agents and attractive smells) [11]. These same compounds possess important health-promoting effects in animals and man; indeed, scientific evidence from clinical trials, epidemiological studies, and in vitro/in vivo tests has demonstrated that a diet rich in specific foods may reduce the risk of many diseases (e.g., cancer, obesity and diabetes, cardiovascular and inflammatory complications) [12].

Foodstuffs are represented by several substances with different nutritional value, biological properties, and structural traits. Some are main or bulk components (e.g., triglycerides or starch), some trace or minor components (e.g., phenolic compounds), and some are pesticides, mycotoxins, or undesirable impurities. The same compound could be a main or minor substance, in relation to the type of considered food [1].

The quantitative and qualitative studies on the characterisation of biomolecules are firstly focused on the selection of good extraction methods [13]. Extraction is the first stage in the study of chemical composition, and it is very important for the outcomes and final results. The main aims of extraction in foods are (i) to extract targeted bioactive or nutritional substances from a complex matrix, (ii) to increase the selectivity of the analytical method, (iii) to increase the sensitivity of the selected test or assay by increasing the quantity of targeted molecules, (iv) to convert the nutritive o active substances into more suitable compounds to be detected and separated, and (v) to allow a more reproducible and strong method that should be not related to variability in the original sample [14]. For this reason, extraction should be carefully performed and optimised. Polarities of different molecules and substances, that may significantly vary because of their relationship with the matrix and their conjugation status, influence the extraction solvents to be selected and used [15]. The selection of solvents for compound extraction should also involve other factors as use of cosolvent, environmental safety, financial feasibility, human toxicity, mass transfer, and molecular affinity between solute and solvent [14]. The optimisation of parameters for compound extraction may improve the extraction efficiency of the molecule of interest and it may also reduce the solvent consumption and waste production in order to allow a more environmentally friendly extraction process [16,17]. Sometimes, specific pretreatment processes are necessary to not modify the raw materials and better extract the substances (e.g., pasteurisation, use of depolymerising enzymes, chemical and microbial acidification storage under specifically modified atmospheres); moreover, additional secondary procedures (e.g., sonication, stirring, rotary shaking, etc.) can be applied to improve the extraction [18]. The main traditional extraction methods are reported in Table 1.

For characterisation (identification and quantification) of nutritional and bioactive molecules, the most important step is the selection of the right analytical strategies to be applied. Spectroscopic protocols usually allow a total identification and quantification of the substances in the food, but in most examples, the specific phytochemical composition may remain not fully observed. In any case, spectrophotometry is usually used for the evaluation of antioxidant capacity [24]. For this reason, more detailed information on the chemical composition requires additional analytical techniques, as chromatography and mass spectrometry, to fully describe the structures of the compounds in the food matrices [14]. Technological improvement and innovation in the analytical instrumentation have allowed more and more sophisticated evaluations (quantitative and qualitative) of the nutritional and health-promoting chemical composition in food [25]. Chromatography is mainly utilised to analyse natural food and relative derived-products. Chromatographic techniques offer very high separation ability because chemical ingredients in a complex extract can be divided into many relatively simple parts (subfractions). Moreover, the last analytical approaches, as the application of hyphenated spectrometry and chromatography (e.g., capillary electrophoresis-diode array detection (CE-DAD), high performance liquid chromatography–diode array detection (HPLC–DAD), gas chromatography/high performance liquid chromatography–mass spectroscopy (GC/HPLC–MS), and high performance liquid chromatography–nuclear magnetic resonance (HPLC–NMR)), may allow to obtain additional spectral information. These techniques are very important for on-line structural elucidation and qualitative analysis [26,27]. In particular, high performance liquid chromatography is now the most widely utilised analytical technique, hyphenated with different detectors (mass spectrometry, fluorescence, and diode arrays) [28]. It is referred as the most important standard strategy in the characterisation and authentication of foodstuffs thanks to its reproducibility, sensitivity, and precision despite large organic solvents consumption, relatively long analytical time, and high instrumental cost [29]. Quality control and product authentication of the food can be carried out by the application of a specific approach based on marker compounds; a marker substance is a chemical ingredient of raw material, preparation, beverage, or food product that is used for characterisation and/or quality control aims, in particular if the nutritive or active constituents are not known or previously identified [30].

Due to this high number of variables, a specific analytical technique is not able to cover all the food analysis aspects. If an analytical method is developed (e.g., for flavonoid analysis), it may be applied for several foods (e.g., soft drinks, fruits, wines, or vegetables). Examples of MS coupled to different chromatographic techniques are shown in the following sections in order to characterise different classes of substances. Some molecules present a closely similar structure (e.g., carbohydrates), while other compounds are defined by their similar biological properties (e.g., vitamins), despite potentially large differences in structure and polarity; for this reason, analytical conditions and sample preparations could be very different depending on the considered molecules.

### 2.1. Proteins

Proteins are a very important dietary ingredient for the survival of humans and animals. They are essential in nutrition to supply enough amounts of specific amino acids, that may be divided into different categories according to their rates of protein in vivo synthesis: (i) indispensable (e.g., valine, tryptophan, threonine, phenylalanine, methionine, lysine, leucine, isoleucine, and histidine); (ii) conditionally indispensable (e.g., tyrosine, cysteine, arginine); (iii) dispensable (e.g., serine, proline, glycine, glutamic acid, glutamine, aspartic acid, asparagine, alanine) [31]. The main studies are focused on physico-chemical traits and structural characteristics of food proteins to elucidate their molecular structure responsible for their actions and functionalities and consequently their relative structure–property relationships. The total nitrogen analysis by Kjeldahl system is the main reference method for the evaluation of proteins in food products and now it is used for validation and/or calibration of alternative methods for protein analysis [32]. Molecules that easily ionise in aqueous solutions (e.g., nitrogen compounds mostly as proteins and single peptides) are potentially suitable for LC–MS techniques. Consequently, LC–MS is fundamental in proteomics and peptidomics, which are branches of biology that assess the full set of peptides or proteins in a single sample. MS-based proteomics and peptidomics are increasingly used for food analysis [33,34].

### 2.2. Lipids

Food derived from plants and animals contain lipids, an essential substance involved in maintaining many life activities. A high number of fat molecules with different functional traits and chemical proprieties are provided by nature. Lipidic molecules significantly contribute to the sensory and nutritional traits of food. Some lipids are structurally simple compounds (e.g., fatty acids), while others show a variable and complex structure (e.g., steroids, sphingomyelins, phospholipids, glycosphingolipids, acylglycerols) [35]. Extract preparation is a crucial step, for analysing lipids and lipid trace substances in lipid-rich systems (e.g., fats derived from animal matrices). HPLC–MS is the main tool used for the characterisation of lipid substances in foods. Two-dimensional HPLC is commonly on-line coupled to MS to improve selectivity for the analysis of single compound structure because of the complexity of these molecules (triacylglycerols, fatty acids, and phospholipids) [36]. In the past, GC–MS techniques were used to analyse triacylglycerols, but this approach required complex derivatisation and extraction procedures. HPLC–MS analysis has been implemented in recent years to simplify sample preparation. The use of MS/MS has enabled MS structural elucidation of individual triacylglycerols [37,38].

### 2.3. Carbohydrates

Carbohydrates are among the most common nutrients in foods and they show many nutritional and physiological roles. For example, celluloses are structural elements in the plant kingdom, and they are defined as an excellent dietary fibre source. Starch and simple sugars (e.g., fructose and glucose) are the main sources of energy in humans and animals. Other carbohydrates are conjugated to several macromolecules, as flavonoids or proteins, and they present several health-promoting and functional roles [39]. It is often very difficult to analyse structural carbohydrates by MS, but these compounds may be targeted by HPLC–MS analysis. Glycoproteins are specialised components of many foodstuffs (e.g., cereals, milk, and colostrum) and possess high functional and nutritional value [40,41,42]. GC–MS, after derivatisation, is traditionally used to analyse small oligosaccharides and monosaccharides. A less common technique for oligosaccharide analysis is MALDI-TOF/TOF; in particular, it is very useful for oligosaccharide structure elucidation, as previously reported by Hotchkiss et al. (2015) [43]. Molecular conjugates and oligosaccharides can be identified and quantified by HPLC–MS, that also allows the analysis of small oligosaccharides and unknown carbohydrates without time-consuming derivatisation, purification, and fractionation. ESI, rather than APCI, is usually preferred as ionisation mode due to the carbohydrate low volatility and high polarity. However, carbohydrates might have low sensitivity in ESI, even if a cationisation may avoid this problem [44].

### 2.4. Vitamins

Health-promoting capacities and nutritional properties of foods were not only associated with the presence of bioactive compounds, as phenolic acids, terpenes, or flavonoids, but they were also attributed to other molecules, such as vitamins. They are very important trace components with several structural features. Today, research in food composition is also involved in the characterisation of antioxidant vitamins (quantity and quality evaluation) and the percentage of their daily requirements. Indeed, thanks to the high vitamin content and the ease of consumption compared to other foods with similar biological properties, consumption of some specific functional foods may be targeted to specific people sectors (e.g., the elderly, sportsmen, pregnant women, and children) [45].

Most of the vitamins are easy to oxidise and thermally-labile [46,47,48] as vitamin C, vitamin B complex, vitamin E, and carotenoids; for this reason, HPLC, in particular if hyphenated to MS techniques, is often used for their identification and quantification. Typically, ESI or, sometimes, APCI ionisation is used when HPLC–MS is employed. Many examples were reported in previous literature for vitamin identification and quantitation in food products by HPLC–MS [49,50] or HPLC-DAD [51,52]. In the last years, HPLC–MS has largely been replaced by ultra-(U)HPLC–MS, providing higher throughput and better chromatographic resolution. Due to their several structural isomeric forms, carotenoids are preferentially analysed by (U)HPLC–MS. APCI (or also atmospheric pressure photoionisation (APPI)) may be more useful than ESI (now commonly used) because most of the carotenoids are apolar compounds [53].

### 2.5. Phenolic Compounds

Phenolics are secondary plant metabolites with many potential health-promoting properties that present a large structural variety. There are many active studies aimed at identifying new chemical molecules and characterising the polyphenolic content of several vegetables, plants, and fruits. Quality and quantity of phenolics in natural foods may significantly vary in accordance with several extrinsic and intrinsic factors (e.g., growing conditions and soil composition, maturity stage, genetics, and post-harvest conditions) [54]. Dietary intake of polyphenolic compounds is greatly influenced by the preferences of single people and eating habits [55]. Simple phenolics (e.g., flavonoids and phenolic acid conjugates) are important ingredients of vegetables, fruits, and relative derived-products as juices, beverages, and jams. These molecules present a wide range of health-promoting properties as the antioxidant capacity and anti-inflammatory activity [56]; for example, they may exert protective effects against cancer, cardiovascular problems, and other major diseases [57].

Polyphenolic compounds may be divided into many several classes [58]; cinnamic acid, benzoic acids, catechins, and flavonols are the most abundant in foods [59,60]. Low quantities of polyphenols are usually observed in complex plant systems and it is very difficult to isolate them in high amounts [61,62]. HPLC–MS is the main techniques for flavonoid analysis with good sensitivity in ESI systems [27]. In particular, MS/MS (or MS^2^) is often utilised in HPLC–MS flavonoids analysis to allow high structural information and mass resolution and increase selectivity after an HPLC-DAD screening [63,64,65].

Regarding structure analysis, the common phenolic fragmentation pathways do not depend on the ionisation mode (APCI, ESI, or matrix-assisted laser desorption ionisation) and the applied analyser. However, the used different instrumentation may significantly influence and change relative fragment abundances [66]. For this reason, it is preferred to detect the presence/absence of specific MS fragments rather than to use relative intensity changes [67].

Information on the glycan part stereochemistry in flavonoid glycosides are not usually provided by HPLC–MS analysis. The sugar part may still be easily identified because glycosidic fragments, commonly present in flavonoid glycosides, show different m/z ions for hexosides (e.g., glucose, galactose), deoxyhexosides (e.g., rhamnose), and pentosides (e.g., xylose, arabinose). Specific MS/MS analysis (MS^n^ spectra) may be used to identify the aglycone in a flavonoid O-glycoside; this result may be also obtained by the comparison with literature data or the corresponding spectra of reference compounds [68]. Enough information for C-glycoside identification may be provided by a specific MS^3^ spectra analysis. Alternatively, the MS/MS aglycone fragments, produced by higher fragmentation energy, may be utilised. Structural characterisation and identification of many flavonoids in different foodstuffs can be achieved by specific mass spectrometric scanning techniques [69].

### 2.6. Allergens

Allergies derived from food are one of the most emergent issues in food science and technology studies. The mainly considered allergens are peanuts, wheat, egg, milk, tree nuts, soybeans, shellfish and fish. In any case, several suspected food allergens have not yet been characterised despite the recent advances in biomedical sciences and immunopathology [1]. The determination of allergens in foods can be carried out by many methods based on the biological immune response, often studied by screening-based strategies, such as enzyme-linked immunosorbent assays (ELISA). However, proteomic techniques and HPLC–MS offer different and unique strengths. The MS high sensitivity and selectivity in protein analysis could overcome some problems associated with appropriate allergen identification [70]. For example, the main peanut allergens in food matrices were successfully identified and quantified at a low ppm level by HPLC–MS/MS [71,72].

### 2.7. Food Additives

Additives in foods are very limited because they should be explicitly authorised before using in foodstuffs. Foodstuffs are monitored for additives and analytical controls are, therefore, an area of increasing importance and concern. Indeed, it is very important to check foodstuffs for additives and HPLC–MS is often the preferred method. Some additives are small compounds (as benzoic acid used for conservation), while other additives are macromolecules; moreover, some additives are synthetic products (composed by a ‘single’ substance), while others are natural extracts (with many and variable components) [1]. Food additives are molecules with different chemical structure grouped according to their application; for this reason, it is difficult to select the best method to be used for their identification and quantification. Most of these analytical strategies are molecule-specific (without an estimation of the total amount of the considered chemical class), while other protocols can be utilised for the characterisation of groups of compounds. Many techniques for the real-time extraction of different agri-food additives and residues in several matrices are used [73].

## 3. Quality and Authentication of Food

Food quality, perceived by consumers, is a critical factor for their final economic value [74]. The consumer often links quality with (i) a specific production system; (ii) the utilisation of particular ingredients; (iii) the food origin (also a particular region); (iv) the product authenticity. The European Union has defined different brands as Protected Geographical Indication (PGI), Traditional Speciality Guaranteed (TSG), and Protected Designation of Origin (PDO) to ensure quality and valorise authentic labelling of food and agricultural products.

Quality is a multifunctional data pattern including physical, chemical, microbiological, technological, and sensorial food traits [75]. Food quality may be defined as “the totality of features and traits of a service or product that bear on its ability to satisfy stated or implied needs” (ISO 9000) [76]. Therefore, quality should not be only addressed by HPLC–MS-based techniques. However, HPLC–MS methods can provide important information on nutritional value, origin, safety, sensory attributes, molecular composition, and unique traits. In the last years, these analytical techniques have become the preferred methods when specific and sensitive molecular characterisation of complex systems is required [77].

The identification of selected molecular markers can be used to assess food quality parameters [78,79]. The utilisation of these biomarker compounds should be defined in terms of quantity or absence/presence, together with their organoleptic, biochemical, and chemical role [80,81]. Moreover, their main metabolic pathway should also be known. Increasing consumer awareness is linked to the development of increasingly sophisticated techniques. Numerous cases of food adulteration have been detected using MS-based techniques [82], as shown in Table 2.

When the aim is to characterise the quality of a specific agri-food production (i.e., cheese, ham, or fruit), several issues should be considered, and many analytical strategies are required. Safety problems to be addressed may include the identification of mycotoxins or agrochemicals, while nutritional traits may require the determination of bulk components (e.g., carbohydrates, proteins, and lipids) plus other minor compounds in trace amounts (e.g., vitamins) [1]. The identification and quantification of compounds with functional or health-promoting properties, as anti-hypertensive peptides or conjugated linoleic acid, is also increasingly required as well as the characterisation of molecular markers for food quality evaluation. The utilisation of chromatographic techniques and relative high separation ability hyphenated to MS molecular identification capability could solve the main analytical problems in food characterisation [67]. Moreover, portable and micro gas-chromatography (Micro GC) is a fast-developing and, in the last years, more mature technologies have been well developed and successfully commercialised with great potential in food and environmental applications [90,91,92]. Multiple Mini MS instruments were also recently developed to be used in food analysis [93].

## 4. Considerations on Statistical Data Analysis

In food analysis, a consistent method should be developed, then optimised, and finally validated by a multivariate statistical experimental design, the Design of Experiments (DoE) [26,94]. In the multivariate statistics, several trials are selected and carried out following a well-designed experimental approach to simultaneously evaluate several factors. The elements that present a specific influence on a chromatographic analytical strategy are defined by a screening design; in food HPLC analysis, these factors may include sample concentration, buffer concentration and relative pH, column temperature and type, mobile phase composition, injection volume, and detection wavelength [95,96].

Moreover, to evaluate the large number of data derived from the hyphenation between chromatographic techniques, as HPLC and GC, and mass spectrometry (e.g., MS, MS^2^, HRMS, etc.), a large range of analytical strategies showed to be versatile and useful tools for the visualisation, extraction, and interpretation of the chemical information. In particular, pattern recognition methods, as cluster analysis (CA) and principal component analysis (PCA), proved to allow a better representation of the information included in the HPLC/GC–MS fingerprints. The original variables are converted into new variables summarising the systemic patterns of the variability among the food samples; indeed, an exploratory data analysis is easier to study if it is shown as a multivariate data table rather than a low-dimensional plane [97].

## 5. HRMS in Food Analysis

For a long time, HRMS techniques have traditionally been limited to the most important applications due to their high instrumental complexity. This situation is now changed thanks to modern HRMS instruments, such as Orbitrap, Fourier-transform ion cyclotron resonance (FT–ICR), and ToF. FT–ICR and Orbitrap MS are high-resolution instruments applied in quality and safety food analysis thanks to their high mass precision (1–2 ppm, high discrimination between ions of interest and isobaric interferences) and high resolution (typically 100,000–1,000,000 FWHM) [98]. In particular, Orbitrap instruments have a resolution higher than 200,000. A recent hybrid mass spectrometer combines a state-of-the-art segmented quadrupole for high-performance precursor ion selection with a high-resolution (HR) and accurate-mass (AM) ultra-high-field Orbitrap mass analyser with a final resolution value of 240,000 [99]. The resolution for these instruments is usually reported at 200 Da and it decreases with the increase of the square root of the *m/z* value and it is proportional to the acquisition time. Instead, TOF instruments have a resolving power that changes very little with the mass [100]. Low-resolution mass analysers show a resolving power of <10,000 (mass accuracy > 5 ppm), while high-resolution ones present a resolving power of 10,000–100,000 (mass accuracy < 5 ppm). In target analysis, specificity may be improved by changing from low to high resolution, but it cannot be clearly defined by comparing LC–HRMS and LC–MS/MS performance [5] and the choice may be more influenced by additional tests, such as retrospective data analysis by LC–HRMS [4].

For many years HRMS coupled to GC has been the main technology used for food analysis. Currently, LC–MS/MS and LC–MS are extensively accepted, and the switch from GC–MS to LC–MS was encouraged by many analysts [101]. Moreover, many commercially available HRMS detectors can be easily hyphenated to LC systems rather than GC systems. LC–MS is preferred to GC–MS thanks to its additional selectivity (due to lower detection limits and less use of an extensive sample clean-up) and its familiarity with LC–MS/MS [102,103].

In the last few years, LC–HRMS has become the most used technique in food analysis, as shown in Table 3, due to a very high number of bioactive compounds and relative derived-degradation products to be analytically monitored by a single protocol [104].

HRMS is a tool for nontargeted or semitargeted screening, that allows to detect suspected peaks and identify and confirm the relative chemical structure. Screening is useful for a no–yes answer in relation to the detection of a set of several molecules or a single compound in many samples. Speed and cost-effectiveness are the main considered parameters. Although quantification may be required, screening analysis is focused on the absence of false-negative data and the number of false-positive findings at specific concentrations [102]. MS techniques (if compared to GC–flame ionisation detection or LC–UV detection) provide a high selectivity that allows verifying lower analyte concentrations in complex matrices without too many false-positive findings. MS/MS-based targeted multiresidue screening techniques allow an even higher selectivity [111,115]. Modern MS/MS instruments are capable of selected reaction monitoring (SRM) at trace concentrations with shorter dwell times (5–10 ms). Therefore, many molecules can be monitored (up to 1000) by the application of retention time-window-based SRM traces and high-end instruments [116]. HRMS is now used as the main screening tool to distinguish between potential target molecules, but without reference standards (unlike LC–MS/MS) [4].

## 6. HRMS Related to Adulteration and Authenticity

Adulteration is the voluntary addition of one or more nonauthorised substances to food products for economic gain. Adulteration is often related to food quality, with significant differences in the price between lower and higher grade food products [117].

Adulteration is closely linked to authenticity [118,119], although authenticity is commonly related to the voluntary mislabelling as economic deception [120]. Rice, honey, olive oil, wine, and fruit-derived products (as jams or juices) are the main products that relate their authenticity to botanical species, floral type, and geographical origin [121,122,123,124]. Table 4 reports data on 137 identified single incidents of economically motivated adulteration in several food categories, based on media reports journal and articles from 1980 to 2013 [120]:

Targeted analyses (i.e., adulterant is known) and untargeted ones (i.e., adulteration involves not known substances) are related to the detection of adulteration of beverages and foods [125]. For example, in dietary supplement analysis, the matrix is already under suspicion because the new adulterant is often structurally linked to an already known molecule or his chemical analogue [126]. Authenticity confirmation is more demanding than adulteration one; for this reason, LC–HRMS can be used as an effective fingerprinting tool, that, coupled to chemometrics, allows to obtain a probability (sometimes a percentage) of authenticity for a foodstuff [4].

In the last years, the use of MS-Orbitrap interfaced with an ion trap mass analyser has continuously increased for fingerprint applications. The developments in chromatography and relative interface techniques and the advances in MS technologies (e.g., hybrid MS instruments composed by two or more mass analysers) will further increase the potential of analytical technologies in food analysis [127].

## 7. Conclusions

In the last years, the agri-food industry started to apply innovative analytical strategies for a full characterisation of food productions because a molecular characterisation was required by regulatory agencies and consumers. These requests shifted the food sector to the pharmaceutical one. Therefore, validated analytical strategies using high-performance systems were improved to ensure food authenticity, safety, and quality. Chromatographic techniques coupled to suitable detection strategies produce an effective tool to separate the single molecules and develop a specific profile of the food sample, called “fingerprint”; the combination of a chromatographic separation system with a spectroscopic detector (mass spectrometry) has become the most used approach for the characterisation and/or confirmation of the identity of selected and/or unknown phytochemical substances. If chromatography, hyphenated with mass spectrometry or other detection systems, is further combined with chemometric techniques, clearer patterns might be developed for analytical fingerprints.

The literature related to MS in the analysis of food quality and safety has recently increased. In this review, a brief overview of the main MS and HRMS application to the food analysis was provided. Indeed, accurate, reliable, and rapid MS techniques for analyte characterisation in food products are indispensable for food quality and safety control. New (U)HPLC–MS(MS) systems allowed to characterise many food products at the molecular level routinely identifying and quantifying desirable and undesirable molecules in different foods. HPLC–MS is often the analytical technique with the highest performance level because it provides a simultaneous assessment of several substances in complex systems (e.g., food products). For this reason, it is now an effective tool for the certification of food authenticity, quality, and safety.

The potential of HPLC–MS is due to the hyphenation of the HPLC separation power and the molecular structure identification provided by MS. The sample-preparation procedures have been minimised and the selectivity for quali-quantitative analyses of complex matrices (e.g., food samples) increased using coupled MS techniques (LC–MS and GC–MS) and MS/MS. Moreover, the developments of HRMS for FT–ICR–MS, Orbitrap, and quadrupole-ToF allowed higher specificity and sensitivity for detection of potential unknown compounds in food (e.g., biomolecules, toxins, and pollutants) and routine food analysis. The analysis of substances with different polarity ranges was allowed by the availability of different ionisation techniques (ESI, APCI, APPI).

Another challenge for food quality and safety control is to promptly provide (often within 1 day) accurate results avoiding damage to food samples. The recently developed ionisation techniques have shown potential, thanks to the ease of automation, fast acquisition, and no-sample treatment. For this reason, the development of reliable, robust, and simple portable MS instruments for accurate, rapid, and in situ characterisation of molecules in food samples remains one of the main future targets. The growing interest in LC-Orbitrap applications for food analysis is associated with important improvements in the data handling software and to reductions in the instrumentation costs. These advances will stimulate high interest in Foodomics for (i) verification of authenticity, (ii) detection of adulteration, (iii) fingerprinting of foods, and (iv) detection of illegal veterinary drugs.

Finally, three main trends could become increasingly important for food analysis: (i) HPLC–MS methods will be further improved and used to solve new issues in the agri-food industry; (ii) analytical protocols will be developed to reduce detection limits for harmful molecules or to improve the multi-compound analysis; (iii) HPLC–MS will be applied in regulatory agencies and food industry, mainly in routine quality control at the production and commercialisation steps.

## Figures and Tables

**Table 1:** Analytical methods for the extraction of the main molecules in foodstuffs [15,19,20,21,22,23].

Method	Used Solvents	Time	The Volume of Requested Solvent (mL)
Accelerated solvent extraction, static (ASE)	Methanol	20–40 min	20–40
Microwave assisted extraction (MAE)	Ethanol, methanol, or a mixture of water and alcohol	10–40 min	20–50
Pressurised hot water extraction (PHWE)	Water with/without 10–30% ethanol	40–50 min	40–45
Pressurised liquid extraction, dynamic (PLE)	Methanol	20–40 min	20–30
Sonication	Ethanol, methanol, or a mixture of water and alcohol	1 h	50–100
Soxhlet extraction	Ethanol, methanol, or a mixture of water and alcohol	3–18 h	150–200
Supercritical fluid extraction (SFE)	Carbon dioxide with/without modifiers (e.g., methanol)	30–100 min	Not applicable
Surfactant assisted PHWE	Water with surfactants (e.g., SDS or Triton X100)	40–50 min	40–45

**Table 2:** Cases of food adulterations solved by the application of mass spectrometry coupled to chromatographic techniques.

Food	Adulteration	Analytical Technique	Reference
Meat, milk	Melamine and its metabolites (cyanuric acid, ammelide, and ammeline) in animal feed, meat, milk and infant formulations, and other processed productions	HPLC–MS/MS	[83]
Food colourants	Harmful colourants as Sudan I–IV dyes (lipophilic azo dyes, used in scientific and industrial applications, even if banned as food colourants because of their carcinogenicity)	HPLC–MS	[84,85,86]
Wine	(i) Addition of sugar even if forbidden; (ii) illegal mixing of different cultivars; (iii) origin falsification; (iv) flavouring or colouring wines by fruit extracts (e.g., elderberry);	HPLC–MS (anthocyanin profiles)	[87,88,89]

HPLC–MS, high performance liquid chromatography–mass spectrometry; HPLC–MS/MS, high performance liquid chromatography–tandem mass spectrometry.

**Table 3:** Use of high-resolution MS in several agri-food analyses.

Analyte	Other Used Methods	Analytical Problems	Mass Spectrometry Methods ^1^	Reference
Marine biotoxins	Mice assay	A high number of false-negative and false-positive findings	LC–MS LC–MS/MS	[105,106]
Mycotoxins	LC ^1^-based detection (e.g., electrochemical Kobra^®^ cell or fluorescence) or immunoassay tests	Need for a good clean-up and high-sensitivity detection and use of expensive and time-consuming but very specific immunoassay	LC–ESI/MS, LC–MS/MS	[107,108,109]
Veterinary drug residues	Immunoassay tests or GC ^1^-fluorescence detection	Need for derivatisation before injection into the GC due to high molecular weight and polarity of these compounds	LC–MS/MS UHPLC–MS/MS	[110,111]
Organic contaminants	GC ^1^-electron capture detection	Low limits of detection and complexity of the matrix	GC–MS/MS LC–MS/MS UHPLC–QToF–MS	[112,113]
Bioactive compounds (e.g., proteins with allergenic potential)	Bioassays (e.g., enzyme-linked immunosorbent assay)	Expensive and time-consuming tests	LC–MS/MS	[114]

^1^ LC–MS, liquid chromatography–mass spectrometry; LC–MS/MS, liquid chromatography–tandem mass spectrometry; LC, liquid chromatography; LC–ESI/MS, liquid chromatography–electrospray ionisation/mass spectrometry; GC, gas chromatography; UHPLC–MS/MS, ultra-high performance liquid chromatography–tandem mass spectrometry; GC–MS/MS, gas chromatography–tandem mass spectrometry; UHPLC–QToF–MS, ultra-high performance liquid chromatography–quadrupole-time of flight–mass spectrometry.

**Table 4:** Incidents of economically motivated adulteration in 11 food categories from 1980 to 2013.

Food Category	Number of Incidents
Seafood and fish	24
Dairy products	15
Fruit juices	12
Fats and oils	12
Grain products	11
Natural sweeteners and honey	10
Herbal extracts and spices	8
Alcoholic beverages and wine	7
Infant products	5
Proteins by plant material	5
Other food products	28
